# The Effects of Jieduquyuzishen Prescription-Treated Rat Serum on the BAFF/BAFF-R Signal Pathway

**DOI:** 10.1371/journal.pone.0118462

**Published:** 2015-02-17

**Authors:** De-hong Wu, Li Xu, Cheng-ping Wen, Guan-qun Xie, Jin-jun Ji, Jie-li Pan, Yi-feng Jiao, Yong-sheng Fan

**Affiliations:** 1 First Clinical Medical College, Zhejiang Chinese Medical University, Hangzhou, China; 2 College of Basic Medicine, Zhejiang Chinese Medical University, Hangzhou, China; Instituto Nacional de Ciencias Medicas y Nutricion Salvador Zubiran, MEXICO

## Abstract

Systemic lupus erythematosus (SLE) is a chronic inflammatory disease mainly characterized by B cell hyperactivity. Glucocorticoid (GC) is widely used in SLE for its potent anti-inflammatory and immunosuppressive effects. Despite its important clinical efficacy, high-dose or long-term use of GC can cause severe side effects, such as osteoporosis, osteonecrosis, cataracts, hyperglycemia, coronary heart disease and cognitive impairment. Our early clinical studies have shown that Jieduquyuzishen prescription (JP) can effectively reduce the adverse effects and improve the curative effect of GC in the treatment of SLE. The BAFF/BAFF-R signaling pathway plays an important role in the development of SLE and has been regarded as a potential target for the therapy of SLE. In this study, we attempt to investigate the effect of JP on the BAFF/BAFF-R signaling pathway to explore the mechanism of JP in reducing the toxicity and enhancing the efficacy of GC. YAC-1 cells, isolated rat peripheral blood lymphocytes, polymorphonuclear neutrophils and spleen lymphocytes were treated with drug-containing serum. The results of RT-PCR, Western blot and dual-luciferase reporter gene assays indicate that either JP or GC can inhibit the mBAFF-induced up-regulation of BAFF, BAFF-R, Bcl-2, IL-10 and NF-κB in YAC-1 cells and WEHI-231 cells. Furthermore, MTS, flow cytometry and CFSE results reveal that the proliferation and survival of lymphocytes activated by mBAFF are suppressed by JP, GC and their combination. Contrary to GC, JP can reduce the apoptosis and raise the survival of polymorphonuclear neutrophils and can’t increase the apoptosis of the peripheral blood lymphocytes and spleen lymphocytes. Therefore, it is possible that JP can down-regulate the BAFF/BAFF-R signaling pathway as effectively as GC, which may result in the dosage reduction of GC, thus decreasing the toxicity and improving the efficacy of GC-based treatment of SLE.

## Introduction

Systemic lupus erythematosus (SLE) is a generalized autoimmune disease featured by immunological dysfunction, involving hyperactivated B cells, abnormally activated T cells, and defective clearance of apoptotic cells and immune complexes [1,2]. A series of autoantibodies, in particular antinuclear antibodies (ANAs), are detected in patients [3]. Immunosuppressants such as glucocorticoid (GC) and hydroxychloroquine sulfate are commonly used drugs for the treatment of SLE [4,5]. GC is a potent anti-inflammatory and immunosuppressive agent that is widely used in SLE. Despite its important clinical efficacy, GC increases the risk of osteoporosis, cataracts, hyperglycemia, coronary heart disease, peptic ulcers and gastrointestinal bleeding [6], thromboembolism [7] and other illnesses, which limits its clinical use; most of these side effects are time- and dose-related. Therefore, reducing the adverse effects and improving the curative effect of GC is important for the treatment of SLE. SLE is considered as a refractory disease that involves complex mechanisms. Thanks to the multi-target, multi-channel characteristics of traditional Chinese medicine (TCM), TCM has a unique role in the treatment of SLE. Jieduquyuziyin prescription (JP), a traditional Chinese medicine prescription, which includes aerial parts of *Artemisia annua L*., rhizome of *Cimicifuga heracleifolia Kom*., dried aerial parts of *Hedyotis diffusa Willd*, et al, is widely used for SLE treatment in China and has been shown to have therapeutic effects [8]. More importantly, JP which has the effects of heat-clearing and detoxifying, activating circulation to remove blood stasis, nourishing Yin and strengthening kidney can reduce the dosage of GC to decrease the side effects and improve efficacy even after long-term use [9–12], but the concrete mechanism remains unclear.

Abnormal B-cell activation and differentiation is associated with the development of SLE. B cell-activating factor (BAFF) has been shown to be a powerful regulator of B cell biology. BAFF has a stimulatory effect on the peripheral B-cell population, and in vitro it can promote the survival of B cells. Excessive BAFF could lead to mature B-cell hyperplasia, driving autoimmune diseases. Overexpression of BAFF has been regarded as an important factor in the pathology of SLE, and levels of BAFF in serum were increased in some SLE patients. Thus, the symptoms of SLE may be improved by down-regulating the BAFF/BAFF-R signaling pathway [13,14].

In this study, we set out to investigate the effect of JP-treated rat serum on the BAFF/BAFF-R signaling pathway to explore the mechanism of JP in reducing toxicity and enhancing the efficacy of GC in the treatment of SLE. The mRNA levels of some key components in the BAFF/BAFF-R signaling pathway, including BAFF, BAFF-R, NF-κB, Bcl-2 and IL-10, were detected by RT-PCR. The protein expression of BAFF-R, NF-κB and phospho-NF-κB were detected by Western blot. The transcriptional activity of NF-κB was measured via a dual-luciferase reporter gene assay. Proliferation was analyzed using the MTS and CFSE methods, and apoptosis of the peripheral blood lymphocytes, polymorphonuclear neutrophils and spleen lymphocytes was evaluated by flow cytometry. The experiments revealed that both JP, GC and their combination can suppress the mBAFF-activated BAFF/BAFF-R signaling pathway in YAC-1 cells and WEHI-231 cells, inhibiting the proliferation and survival of YAC-1 cells and lymphocytes. These data suggest that JP can inhibit the BAFF/BAFF-R signaling pathway, which may be associated with the mechanism of the JP-mediated reduction of GC dosage for the treatment of SLE.

## Materials and Methods

### Animals and Cells

The experimental protocol was approved by the Animal Welfare Committee of Zhejiang Chinese Medical University, Hangzhou, China (SYXK2008–0115). The animal housing facility is a barrier housing facility, and it is maintained in accordance with the national standards (Laboratory Animal-Requirements of Environment and Housing Facilities, GB 14925–2001). Sprague-Dawley (SD) rats (n = 38, weighing 200±20 g, 28 males and 10 females) were obtained from the Laboratory Animal Research Center of Zhejiang Chinese Medical University. YAC-1 cells and WEHI-231 were purchased from the Shanghai Institutes for Biological Sciences, Chinese Academy of Sciences.

### Reagents

The raw herbal medicines for JP preparation were purchased from the Chinese Herbal Medicine Co., Ltd. of Zhejiang Chinese Medical University (Hangzhou, China). RPMI-1640 culture medium (No. 11875093), fetal bovine serum (FBS, No. 10100–147), Lipofectamine 2000 (No. 11668) and Trizol reagent (No. 15596–026) were purchased from Invitrogen. The PrimeScript RT-PCR kit (DRR014S), TaKaRa Taq (DR001A) and 100 bp DNA Ladder (3422A) were purchased from TaKaRa company, Japan. Recombinant mouse BAFF (mBAFF, No. 2106-BF) was purchased from R&D Systems. Dexamethasone (DEX, No. D1756) was purchase from Sigma, USA. The Bradford Protein Assay Kit (No. P0006) was purchased from Beyotime Inst. Biotechnology, China. The Dual-Luciferase reporter assay system kit (No. E1910) and Cell Titer 96 AQueous One Solution Cell Proliferation Assay (MTS, No. G3582) were purchased from Promega, USA. Rat peripheral blood lymphocyte separation medium kit (No. LTS1083) and rat polymorphonuclear neutrophil separation medium kit (No. LZS1091) were purchased from Tianjin Haoyang Biological Manufacture Co., Ltd., China. Cell Trace CFSE cell proliferation Kit (Life technologies C34554).

### Primary and Secondary antibodies

BAFF-R (No. SC-19887) and NF-κB antibodies (No. SC-372) were purchased from Santa Cruz, USA. The pS536-NF-κB antibody (No. 3033) and GAPDH antibody (No. 5174) were purchased from Cell Signaling Technology. The goat anti-rabbit RDye 680 secondary antibody (No. 926–32221) and goat anti-mouse RDye 800 secondary antibody (No. 926–32210) were purchased from LI-COR Biosciences, USA.

### JP Preparation

The JP formula used in the experiment was composed of 10 herbs, including dried aerial parts of *Artemisia annua L*., rhizome of *Cimicifuga heracleifolia Kom*., dried aerial parts of *Hedyotis diffusa Willd*, prepared root of *Paeonia veitchii Lynch*, carapace of *Trionyx sinensis Wiegmann*, dried aerial parts of *Centella asiatica* (Linn.) Urban, fruit of *Citrus medica Linn*. *var*. *sarcodactylis* (Noot.) Swingle, rhizome of *Glycyrrhiza uralensis Fisch*, seed of *Coix lacryma-jobi L*.*var*.*mayuen* (Roman.) Stapf, prepared root of *Rehmannia glutinosa* (Gaert.) Libosch.. Traditional Chinese medicine of the above-mentioned herbs were crushed and mixed together at a ratio of 5:4:4:9:5:4:5:5:3:2. After soaking in water (w/v, 1/10) for 1 h, the mixed herbs were boiled for 2 h for extraction. The residue was extracted again for another 2 h. The filtrates were collected, combined and concentrated to 0.5 g, 1.0 g, 1.5 g crude drug/mL.

### JP-treated Rat Serum Preparation

Male SD rats weighing 200±20 g were divided into JP groups (low, moderate and high doses) and control groups. JP groups were administrated with different doses of JP or normal physiological saline via gastrogavage respectively, 5 mL/kg, twice a day, for 3 days. One hour after the last administration, the blood of the JP group and control group were sterilely collected separately through the celiac vein. After settling for 3–4 h at room temperature, the JP-treated rat serum and the blank serum were separated by centrifugation at 3000 r/min at 4°C for 20 min, and then stored at -70°C after inactivating at 56°C for 30 min.

### YAC-1 Cell, WEHI-231 Cell Culture and Treatment

YAC-1 cells and WEHI-231 cells were cultured in RPMI-1640 containing 10% fetal bovine serum in a 5% CO2 incubator (Heraeus Holding GmbH, Germany) at 37°C and rinsed twice with minimum essential medium (MEM) (Invitrogen, USA) before testing.

After culture for 24 to 48 h, the YAC-1 cells were divided into 4 groups: 10% blank-control serum (C), 10% blank-control serum plus mBAFF (R), 10% blank-control serum plus mBAFF and DEX (G) and 10% moderate-dose JP-treated rat serum plus mBAFF (J).

WEHI-231, a murine B lymphocyte, was used for the evaluation of dose-dependent effect of JP. WEHI-231 cells were divided into 5 groups treated with 10% blank-control serum group (C), 10% blank-control serum plus mBAFF group (R), 10% low-dose JP-treated rat serum plus mBAFF group (L), 10% moderate-dose JP-treated rat serum plus mBAFF group (M) and 10% high-dose JP-treated rat serum plus mBAFF group (H). Furthermore, the time-dependent effect of JP was investigated with moderate-dose JP-treated rat serum. WEHI-231 cells were treated with moderate-dose JP-treated rat serum for 3 h, 6 h, 12 h and 24 h independently.

After culture for 1 h, the cells in groups which were demanded plus mBAFF were treated with mBAFF 0.1 μg/mL, and cells in groups which were demanded plus DEX were treated with DEX 40 ng/mL. Twenty-four hours later, the cells, protein and RNA were collected without rinsing for further assays.

### Isolation and Treatment of rat Spleen Lymphocytes

Female SD rats weighing 200±20 g were paunched to get spleens under 1% chloral hydrate anesthesia, and cut spleens into pieces, washing it with RPMI-1640 culture medium, centrifuged for 5min at 1500r/min after filtering it with 100 mesh nylon mesh, the supernatant was discarded, and the spleen cells were suspended with RPMI-1640 culture medium, then rat spleen lymphocytes were isolated and collected by using a rat peripheral blood lymphocyte separation medium Kit. Cells were divided into C, R, G, J and 10% moderate-dose JP-treated rat serum plus mBAFF and DEX group(G+J).

### BAFF, BAFF-R, NF-κB, Bcl-2 and IL-10 mRNA Levels Determined by RT-PCR

Total RNA was extracted from the cell layer of each group with Trizol reagent. The quantity and purity of RNA were determined by measuring the absorbance at 260 and 280 nm using a spectrophotometer (Unico Co., USA). The total RNA was reverse transcribed into complementary DNA (cDNA) with a cDNA synthesis kit and amplified in a PCR machine (Eppendorf, Germany). The specific primers for the target genes and β-actin (synthesized by Shanghai Shenggong Co.) were used as described in Table 1.

**Table 1:** Primers for the Target Genes and β-actin Used in RT-PCR.

Target gene	Primer	Length of Target fragment (bp)
β-actin	5’-ATCCGTAAAGACCTCTATGCCAACA-3’(F), 5’-GCCGTGGAGTACGACAA-3’(R)	405
BAFF	5’-TCGTGGAATGGATGAGTC-3’(F), 5’-CTGGTCCCTGGAAAGC-3’(R)	401
BAFF-R(1)	5’-AGAAACTGCGTGTCCTGT-3’(F), 5’-GGTTCCAGCCTCCACT-3’(R)	556
BAFF-R(2)	5’-ATGGCTCAGCAGTTCG-3’(F), 5’-GCTATGTAGACCAGGATGG-3’(F)	452
NF-κB	5’-GCCGTGGAGTACGACAA-3’(F), 5’-CGGTTTCCCATTTAGTATGT-3’ (R)	345
Bcl-2	5’-GCCTCTTCACCTTTCAGC-3’(F), 5’-GCATCCCACTCGTAGCC-3’(R)	406
IL-10	5’-TACTGCTAACCGACTCCTTA-3’(F), 5’-TTCATGGCCTTGTAGACAC-3’(R)	286

A two-step PCR procedure was recommended as follows: pre-denaturation for 10 s at 94°C, 1 cycle; 94°C for 30 s, 54°C for 30 s and 72°C, 10 min, 30 cycles. The final products were identified by electrophoresis on a 1% agarose gel and analyzed with an automatic image analyzer. The mRNA level of β-actin was used as an internal control, and gene-specific mRNA expression was normalized against β-actin expression. Data are presented as mean±SD of triplicate measurements

### Protein Expression of BAFF-R, NF-κB and phospho-NF-κB in YAC-1 Cells Determined by Western Blot

Total protein was extracted using Beyotime Lysis Buffer (Beyotime, China) and analyzed with a bicinchoninic acid (BCA) protein assay kit. The sample proteins were separated by electrophoresis on a 10% SDS-PAGE separating gel with the Bio-Rad electrophoresis system, then transferred to a polyvinylidene difluoride (PVDF) membrane. The transferred membranes were incubated with primary antibodies (GAPDH antibody, 1:5000 dilution; BAFF-R antibody, 1:1000 dilution; NF-κB antibody, 1:1000 dilution; phospho-NF-κB, 1:1000 dilution), then exposed to the corresponding secondary antibody (1:10000 dilution). The protein bands were visualized using the Odyssey Infrared Imaging System (LI-COR Biosciences) and the bands were quantitated with Odyssey Infrared Scanning system software. A GAPDH protein antibody was used as an internal control. Target protein expression was normalized against GAPDH protein expression. Data are presented as mean±SD of triplicate measurements.

### Apoptosis Analysis of Rat Peripheral Blood Lymphocytes, Polymorphonuclear Neutrophils and Spleen Lymphocytes by Flow Cytometry

The isolated peripheral blood lymphocytes, polymorphonuclear neutrophils and spleen lymphocytes were divided into four groups (C, R, G and J), spleen lymphocytes were divided into 5 groups (C, R, G, J and G+J). The cells were washed twice with cold PBS and then resuspended in 1 x Binding Buffer at a concentration of 1 x 10^6^ cells/ml. One hundred microliters of the solution(1 x 10^5^ cells)were transferred into a 5 ml culture tube, then 5 μl of Annexin V-FITC and 5 μl of Propidium Iodide (PI) were added. The cells were gently vortexed and incubated for 15 min at room temperature in the dark. Next, 400 μl of 1x Binding Buffer were added to each tube. The prepared samples were analyzed by flow cytometry (BECKMAN CYTOMICS FC 500) within 1 hour.

A double-parameter map was made showing FITC and PI fluorescence intensity. The cells were divided into four subgroups in the two-dimensional FCM map: left lower quadrant (B3), Annexin V–FITC–/PI– for the living cell group (LC); right lower quadrant (B4), Annexin V–FITC+/PI– for the viable apoptotic cell group (VA); right upper quadrant (B2), Annexin V—FITC+/PI+ cells for the non-viable apoptotic cell group (NVA); upper left quadrant (B1), AnnexinV—FITC–/PI+ for dead cells and other debris (DC).

### Transcriptional Activity of NF-κB in YAC-1 Cells Detected by Dual-luciferase Reporter Gene Assay

Experimental constructs were transfected into YAC-1 cells using a liposome-mediated method and Lipofectamine 2000 (Invitrogen, USA). YAC-1 cells with 500 μL fresh culture medium were plated on 24-well plates at a density of 1 × 10^5^ cells per well 1 hour prior to transfection. The NF-κB expression plasmid (0.6 μg per well), NF-κB firefly luciferase reporter plasmid (0.6 μg per well) and the internal control vector pRL-SV40 containing the Renilla firefly reporter gene (0.06 μg per well) were co-transfected into all cells using Lipofectamine 2000 transfection reagent. Twelve hours post-transfection, the cells were divided into 4 groups (C, R, G and J) as described in the previous section. Twenty-four hours later, the transfected cells were lysed and the fluorescence intensity of firefly and Renilla luciferase in the cell lysates was measured by a dual-luciferase reporter assay system. The transcriptional activity of NF-κB in the above 4 groups was expressed as the ratio of firefly to Renilla luciferase abundance (Fluc/Rluc).

### Proliferation Assay of YAC-1 Cells and Rat Peripheral Blood Lymphocytes and Polymorphonuclear Neutrophils Using the MTS Method

YAC-1 cells were seeded at a density of 2,500 cells per well in 96-well plates and allowed to grow to 80% confluency. After 24 hours, the medium was replaced and the cells were divided into 4 groups. The cells were cultured with 10% blank-control serum (group C, R and G), or 10% JP-treated rat serum (group J). After treatment with mBAFF (group R and J) or mBAFF plus DEX (group G) for 24 h, MTS (20 μL/well) was added.

Peripheral blood lymphocytes and polymorphonuclear neutrophils from 6 female SD rats were isolated and collected by using a rat peripheral blood lymphocyte separation medium kit and a rat polymorphonuclear neutrophil separation medium kit. The cells were also divided into four groups (C, R, G and J) to observe proliferation. The cells were incubated at 37°C for 4 h after the addition of MTS (20 μL/well). The absorbance was subsequently measured at 490 nm using an automatic microplate reader (Thermo Fisher Varioskan Flash 3001).

### Proliferation Assay of Rat Spleen Lymphocytes Using CFSE Method

Rat spleen lymphocytes cells were washed twice with cold PBS and then resuspended with RPMI-1640 culture medium without serum at a concentration of 10^7^ cells/ml. Then 5 μl CFSE (5 mmol/L) was added into cells and its final concentration is 5 μmol/L. The cells were gently vortexed and incubated for 15min at room temperature in the dark. Next, the same volume of calf serum was added into cells for 10min to terminate reaction and then washed twice with PBS. CFSE labeled cells were collected and divided into 5 groups (C, R, G, J and G+J) and cultured with RPMI-1640 culture medium. 72 hours later, the cells were collected and washed with PBS twice, the proliferation dynamics of CFSE labeled cells were analyzed by flow cytometry (BECKMAN CYTOMICS FC 500) within 1 hour. The B region of the cells was showed as groups of proliferation cells.

### Statistical Analysis

All values were expressed as the mean ± standard deviation. One-Way ANOVA was used for comparisons between multiple groups (>2). Comparisons between 2 groups was done with *t*-test or Mann—WhitneyU test. Differences were considered statistically significant if the *p* value was less than 0.05. Statistical analysis was carried out by SPSS 16.0.

## Results

### Effect of JP on the mRNA Expression of BAFF, BAFF-R, NF-κB, Bcl-2 and IL-10 in YAC-1 Cells

**Fig 1:**
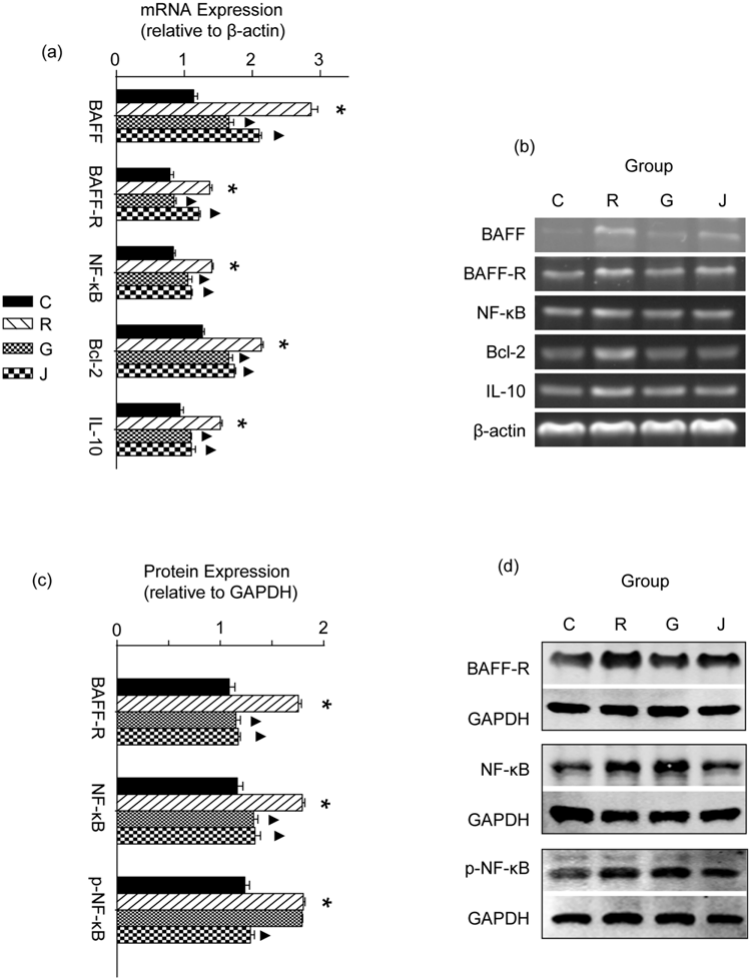
Effect of JP on the mRNA sxpression of BAFF, BAFF-R, NF-κB, Bcl-2 and IL-10 in YAC-1 Cells. The mRNA expression was determined by RT-PCR, the mRNA level of β-actin was used as an internal control, and gene-specific mRNA expression was normalized against β-actin expression (a and b). The protein expression of BAFF-R, NF-κB and phospho-NF-κB in YAC-1 Cells was determined by Western blot (c and d). A GAPDH protein antibody was used as an internal control. Target protein expression was normalized against GAPDH protein expression. C represents the 10% blank-control serum group, R represents the 10% blank-control serum plus mBAFF group, G represents the 10% blank-control serum plus mBAFF and GC group and J represents the 10% moderate-dose JP-treated rat serum plus mBAFF group. Results are shown as means ± SD. * *p*<0.05 compared with the C group, ▲*p*<0.05 compared with the R group.

After stimulation with mBAFF for 24 h, the mRNA expression of BAFF, BAFF-R, NF-κB, Bcl-2 and IL-10 in cells increased significantly in the R group compared to the C group (*p*<0.05). The mRNA expression of BAFF, BAFF-R, NF-κB, Bcl-2 and IL-10 decreased significantly in the G group under the intervention of GC (*p*<0.05). A similar phenomenon was also found in the J group, indicating that JP plays a similar role as GC in regulating the mRNA expression of BAFF, BAFF-R, NF-κB, Bcl-2 and IL-10 (*p*<0.05, Fig. 1a and b).

### Effect of JP on Protein Expression of BAFF-R, NF-κB and phospho-NF-κB in YAC-1 Cells

After stimulation with mBAFF for 24 h, the protein expression of BAFF-R, NF-κB and phospho-NF-κB in YAC-1 cells increased significantly in the R group compared to the C group (*p*<0.05). The protein expression levels of BAFF-R and NF-κB were significantly lower in the G group than in the R group (*p*<0.05), but the phospho-NF-κB level did not change significantly (*p*>0.05). Compared with the R group, the protein expression of BAFF-R, NF-κB and phospho-NF-κB decreased significantly in the J group (*p*<0.05, Fig. 1c and d).

### Doses-dependent Effect of JP on mRNA Expression of BAFF-R, NF-κB, Bcl-2 and IL-10 in WEHI-231 Cells

**Fig 2:**
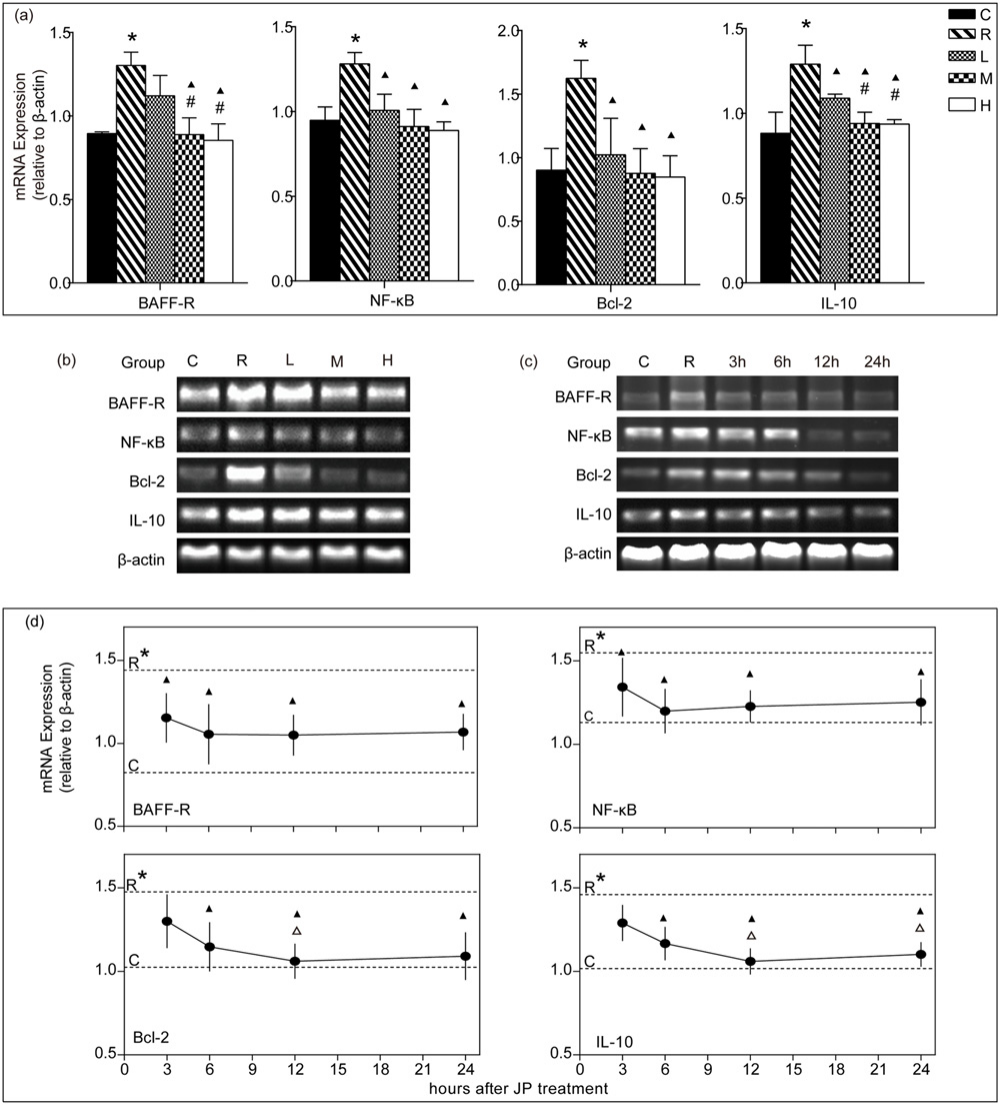
mRNA expression of BAFF-R, NF-κB, Bcl-2 and IL-10 in WEHI-231 cells treated with JP at different doses (a and b) and different times (c and d). The mRNA level of β-actin was used as an internal control, and gene-specific mRNA expression was normalized against β-actin expression. C represents the 10% blank-control serum group, R represents the 10% blank-control serum plus mBAFF group, L represents the 10% low-dose JP-treated rat serum plus mBAFF group, M represents 10% moderate-dose JP-treated rat serum plus mBAFF group, H represents 10% high-dose JP-treated rat serum plus mBAFF group. 3h, 6h, 12h and 24h means that cells were treated with moderate-dose JP-treated rat serum for 3 hours, 6 hours, 12 hours and 24 hours respectively. Results are shown as means ± SD. * *p*<0.05 compared with the C group, ▲ *p*<0.05 compared with the R group, **#** *p*<0.05 compared with the L group, △ *p*<0.05 compared with the 3h group.

After stimulation with mBAFF for 24 h, mRNA expressions of BAFF-R, NF-κB, Bcl-2 and IL-10 in cells increased significantly in R group compared to the C group (*p*<0.05). Compared with the R group, mRNA expressions of NF-κB, Bcl-2 and IL-10 decreased (*p*<0.05), but the BAFF-R did not change significantly in L group (*p*>0.05); mRNA expressions of BAFF-R, NF-κB, Bcl-2 and IL-10 decreased significantly in M and H groups (*p*<0.05). NF-κB and Bcl-2 had no significant difference between the L, M and H groups (*p*>0.05). BAFF-R and IL-10 was significantly lower in M and H groups than in L group (*p*<0.05), but BAFF-R and IL-10 did not change significantly between the M and H groups (*p*>0.05, Fig. 2a and b).

### Time-dependent Effect of JP on mRNA Expression of BAFF-R, NF-κB, Bcl-2 and IL-10 in WEHI-231 Cells

After stimulation with mBAFF for 24 h, mRNA expressions of BAFF-R, NF-κB, Bcl-2 and IL-10 in cells increased significantly in R group as compared to the C group (*p*<0.05). Compared with the R group, mRNA expressions of BAFF-R, NF-κB decreased significantly in 3h group(*p*<0.05), and BAFF-R, NF-κB, Bcl-2 and IL-10 decreased significantly in 6h, 12h, 24h groups (*p*<0.05), but Bcl-2 and IL-10 did not changed significantly in 3h group (*p*>0.05). Bcl-2 in 12h group and IL-10 in 12h and 24h groups decreased significantly compared to 3h group(*p*<0.05), but BAFF-R and NF-κB had no significant difference in 3h, 6h, 12h groups compared to the 24h group (*p*>0.05, Fig. 2c and d).

### Effect of JP on Apoptosis in Rat Peripheral Blood Lymphocytes, Polymorphonuclear Neutrophils and Spleen Lymphocytes

**Fig 3:**
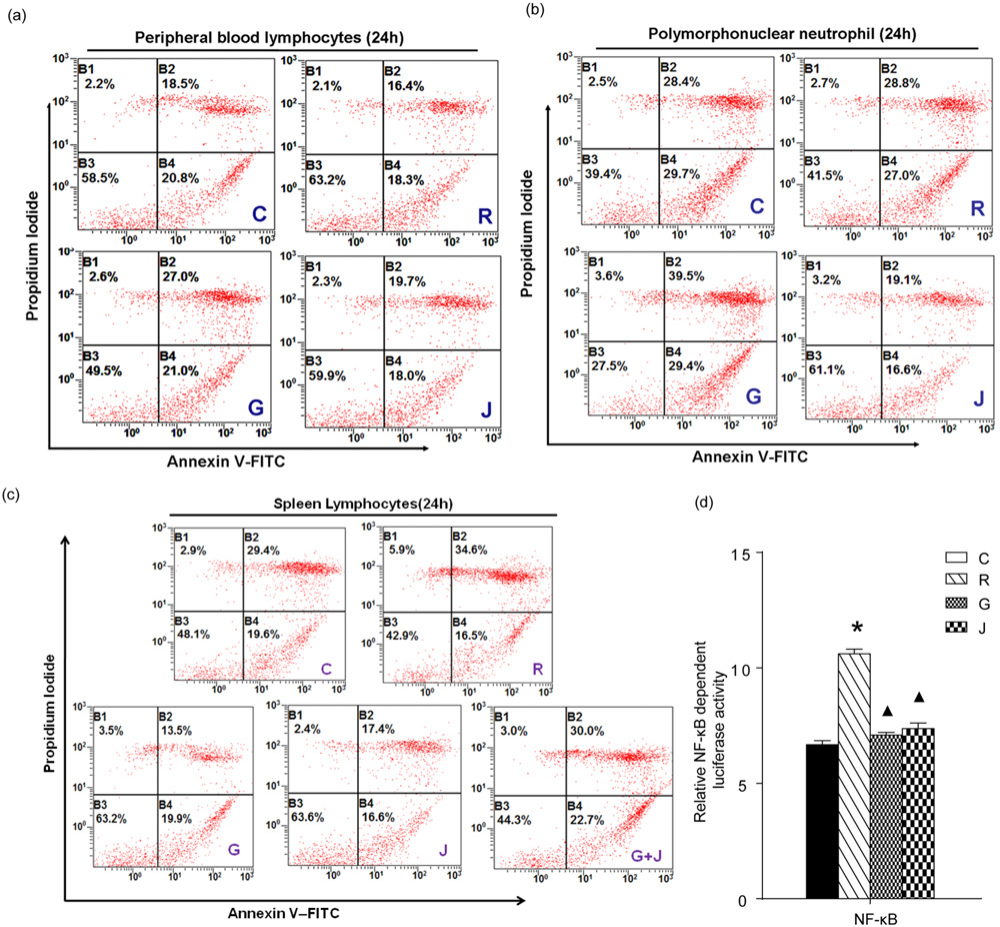
Effect of JP on the apoptosis of rat peripheral blood lymphocytes (a), polymorphonuclear neutrophils (b), spleen lymphocytes (c) and on the transcriptional activity of NF-κB in YAC-1 cells (d). The percentage of apoptotic cells was assessed by flow cytometry as described. The blue characters in the lower right part of each figure represent different treatment groups. The transcriptional activity of NF-κB was detected by dual-luciferase reporter gene assay and expressed as the ratio of firefly to Renilla luciferase abundance (Fluc/Rluc). Data is shown as means ± SD. *p<0.05 compared with the C group, ▲*p*<0.05 compared with the R group. C represents the 10% blank-control serum group, R represents the 10% blank-control serum plus mBAFF group, G represents the 10% blank-control serum plus mBAFF and GC group, J represents the 10% moderate-dose JP-treated rat serum plus mBAFF group.

The influence of JP-treated rat serum on the apoptosis and survival of rat peripheral blood lymphocytes, polymorphonuclear neutrophils and spleen lymphocytes was measured by flow cytometry. The result was derived from a single experiment and didn’t make statistical analysis. As shown in Fig. 3, a, b and c, the apoptotic ratio decreased significantly in peripheral blood lymphocytes, polymorphonuclear neutrophils and spleen lymphocytes after stimulation with mBAFF for 24 h. When treated with GC (G group), the apoptotic cells increased significantly in peripheral blood lymphocytes, polymorphonuclear neutrophils and spleen lymphocytes compared to the R group. When treated with moderate-dose JP-treated rat serum for 24 h (J group), the apoptotic cells didn’t increase significantly in both peripheral blood lymphocytes and spleen lymphocytes, and the apoptotic rate of the polymorphonuclear neutrophils decreased significantly compared to the R group. However, the apoptotic ratio increased significantly in spleen lymphocytes when treated with JP-treated rat serum plus DEX (G+J group).

### Effect of JP on the Transcriptional Activity of NF-κB in YAC-1 Cells

After stimulation with mBAFF for 24 h, the transcriptional activity of NF-κB in YAC-1 cells increased significantly in the R group compared to the C group (*p*<0.05). The transcriptional activity of NF-κB was reduced significantly in the G group upon the addition of GC to the culture (*p*<0.05). The same phenomenon was also observed in the J group (*p*<0.05, Fig. 3d).

### Effect of JP on the Proliferation of YAC-1 Cells and Rat Peripheral Blood Lymphocytes, Polymorphonuclear Neutrophils and Rat Spleen Lymphocytes

**Fig 4:**
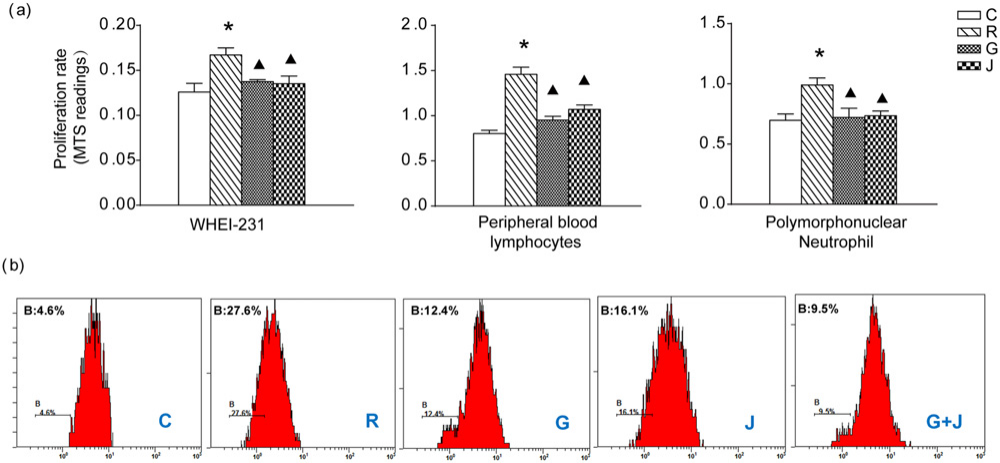
Effect of JP on the proliferation of cells. (a) The proliferation of YAC-1 cells, rat peripheral blood lymphocytes and polymorphonuclear neutrophils determined by MTS. The absorbance was subsequently measured at 490 nm using an automatic microplate reader. (b) The proliferation of rat spleen lymphocytes determined by CFSE method. The proliferation dynamics of CFSE labeled cells were analyzed by flow cytometry within 1 hour. The B region of the cells was showed as group of proliferation cells. * *p*<0.05 compared with the C group, ▲ *p*<0.05 compared with the R group.

The influence of JP on the proliferation of YAC-1 cells, rat peripheral blood lymphocytes and polymorphonuclear neutrophils was assayed by using the MTS method (Fig. 4a). After stimulation with mBAFF for 24 h, the proliferation of these three types of cells was promoted significantly in the R group (*p*<0.05). Compared to the R group, the proliferation was inhibited in both the G and the J groups (*p*<0.05). The influence of moderate-dose JP-treated rat serum on the proliferation of rat spleen lymphocytes was assayed using the CFSE method (Fig. 4 b). After stimulation with BAFF for 72 h in R group, the proliferation cells increased significantly. Compared with the R group, when treated with GC or JP, the proliferation cells decreased obviously in G or R group. In addition, the proliferation cells increased significantly in spleen lymphocytes when treated with the combination use of JP and GC (G+J group).

## Discussion

Abnormal B cell activation and differentiation is a prominent feature of SLE [15]. BAFF was identified as a novel tumor necrosis factor (TNF) family ligand and has proven to be a powerful regulator of B cell biology. The binding of BAFF to BAFF-R can promote the proliferation and survival of B cells [16–20]. BAFF synthesis occurs in several types of cells, including monocytes, activated neutrophils, T/B cells and dendritic cells [21–23]. The BAFF/BAFF-R signaling pathway plays an important role in the pathology of SLE and has been regarded as a potential target for the treatment of SLE.

Mouse models have confirmed that the deficiency of either BAFF or BAFF-R may severely affect B-cell development, and the overexpression of BAFF triggered an elevated number of B cells and results in the development of an autoimmune condition similar to SLE [24–27]. Our study is consistent with these animal studies. It has been demonstrated that after treatment with mBAFF, the levels of BAFF and BAFF-R were both increased in YAC-1 cells and WEHI-231 Cells, and cell proliferation was promoted; in contrast, exposure to JP, GC or their combination down-regulated the expression of these proteins to reduce the activity of the BAFF/BAFF-R signaling pathway, and cell proliferation was disrupted accordingly, this is consistent with previous reports which revealed that GC can inhibit BAFF expression in fibroblast-like synoviocytes from patients with rheumatoid arthritis [28] and the proliferation of lymphocytes in patients with immune thrombocytopenia in vitro experiment [29].

In addition, dose- and time-dependent experiment showed that treatment with high-dose JP for 24h on WEHI cells may play a good effect in suppressintg the activity of the BAFF/BAFF-R signaling pathway.

NF-κB is a critical transcription factor for BAFF-mediated B cell survival and development [30,31]. The results of this study demonstrate that mBAFF treatment not only enhance the mRNA and protein expression of NF-κB but also raise the NF-κB transcriptional activity. In contrast, treatment with JP or GC effectively decreased the levels of NF-κB and inhibited its transcriptional activity, which may cause the suppression of the BAFF/BAFF-R signaling pathway.

NF-κB can promote the up-regulation of several Bcl-2 family members, and Bcl-2 plays an important role in the regulation of apoptosis [32]. Animal models have proven that BAFF/BAFF-R interactions have profound effects on the maturation and survival of B cells [25,26,33]. Deficiency in BAFF-R resulted in a reduction of the B-cell population and a shortening of the cell lifespan; these defects could be rescued by Bcl-2 overexpression, which led to an increase in cell apoptosis [34–37]. It is suggested that BAFF/BAFF-R interactions may promote B cell survival by preventing apoptosis via Bcl-2 [15]. In our research, RT-PCR revealed that exogenous BAFF elevated the mRNA levels of Bcl-2 in YAC-1 cells and WEHI-231 cells, while this effect was inhibited by JP combined with GC, driving an increase in cell apoptosis.

In SLE patients, the binding of BAFF to BAFF-R can induce the production of IL-10, which boosts the generation of autoantibodies in peripheral blood mononuclear cells [38]. The mRNA level of IL-10 was increased in YAC-1 cells and WEHI-231 cells after mBAFF stimulation, while JP and GC prevented the increase in IL-10 to reduce the development of autoantibodies.

After stimulation with mBAFF, the up-regulated levels of BAFF, BAFF-R, NF-κB, Bcl-2 and IL-10 resulted in the significantly increased proliferation of YAC-1 cells, rat peripheral blood Lymphocytes, and spleen lymphocytes; after treatment with JP or GC, all proliferation tended to decrease with the down-regulation of BAFF, BAFF-R, NF-κB, Bcl-2 and IL-10. Moreover, the exposure to JP, GC or JP combined with GC contributed to the inhibition of cell proliferation and survival in lymphocytes.

Additionally, while GC increased mBAFF-stimulated cell apoptosis in polymorphonuclear neutrophils, JP significantly decreased cell apoptosis. It has been suggested that GC and JP have opposite effects on the survival of polymorphonuclear neutrophils. Some studies have shown that neutrophils might play a pathogenic role in the development of SLE [39,40]. In SLE patients, the numbers of apoptotic neutrophils in the blood were increased, which is possibly associated with the disease activity and antibodies against double-stranded DNA [41,42]. The dysregulation of neutrophil apoptosis could lead to the development of SLE. It is conceivable that JP can prevent mBAFF-induced neutrophil apoptosis, which may be related to the therapeutic effect of TCM during SLE treatment. In the study, GC increased the apoptosis of mBAFF-stimulated peripheral blood lymphocytes and spleen lymphocytes, and it is consistent with previous reports that GC was shown to trigger apoptosis of splenic B lymphocytes and ileal Peyer’s. patch B cells [43,44]. It is also found that JP didn’t increase mBAFF-stimulated cell apoptosis in both peripheral blood lymphocytes and spleen lymphocytes. However, the apoptotic ratio increased in lymphocytes when treated with the combination of GC and JP, it is supposed that JP couldn’t increase lymphocytes apoptosis directly but may have an effect on the glucocorticoid receptor(GR) which is a critical factor in GC-induced cell apoptosis [45–47]. Here, the expression of GR is probably raised by JP to enhance the effect of GC to promote cell apoptosis.

Our study tried to explore the mechanism of TCM in SLE therapy from a new perspective of involving the BAFF/BAFF-R signaling pathway, which is implicated in the pathology of autoimmune diseases. It was confirmed that GC and JP have the same inhibitory effects on the BAFF/BAFF-R signaling pathway in YAC-1 cells and WEHI-231 cells. Also, GC, JP or JP combined with GC can inhibit mBAFF-induced cell proliferation and promote the apoptotic ratio in peripheral blood lymphocytes and spleen lymphocytes. The down-regulation effect of JP on the BAFF/BAFF-R signaling pathway may be an important mechanism in reducing the dosage of GC when they are used in combination to treat SLE; thus, the side effects of GC can be greatly diminished.

However, we must acknowledge the limitation of this study. In the Materials and Methods section, Jieduquyuzishen Prescription (JP) was extracted into water. Yet, saline was used as vehicle control for rat gavage, and sera purified from the animals were used for cell culture experiments, which may affect the results of the experiments to some extent.
